# Acinetobacter baumannii Carbapenemase Producers in Morocco: Genetic Diversity

**DOI:** 10.7759/cureus.43629

**Published:** 2023-08-17

**Authors:** Abdelhamid Massik, Lahbib Hibaoui, Moussa Benboubker, Ghita Yahyaoui, Bouchra Oumokhtar, Mustapha Mahmoud

**Affiliations:** 1 Biomedical and Translational Research Laboratory, Faculty of Medicine and Pharmacy, Sidi Mohamed Ben Abdellah University, Fez, MAR; 2 Human Pathology, Biomedicine and Environment Laboratory, Faculty of Medicine and Pharmacy, Sidi Mohamed Ben Abdellah University, Fez, MAR; 3 Microbiology and Molecular Biology Laboratory, Faculty of Medicine and Pharmacy, Sidi Mohamed Ben Abdellah University, Fez, MAR

**Keywords:** carbapenemase genes, oxacillinases, metallo-β-lactamases, carbapenemases, acinetobacter baumannii, carbapenems

## Abstract

Introduction: Carbapenem resistance in *Acinetobacter baumannii* (*A. baumannii*) is a public health problem worldwide. Although carbapenem resistance is emerging in Morocco, few studies have shown the epidemiological profile of carbapenemase genes in Moroccan healthcare facilities. The aim of this study was to characterize the molecular profile of the carbapenemase enzyme in *Acinetobacter baumannii* from clinical isolates.

Methods: Clinical strains isolated in the laboratory from various samples were subjected to several phenotypic tests. Antibiotic susceptibility and identification were tested using Phoenix 100 (Becton Dickinson Co., Sparks, MD, USA) and Api 20 (bioMérieux, Marcy-l'Etoile, France). Simple phenotypic assays were used to detect carbapenemase oxacillinase (OXA) and metallo-β-lactamase (MBL) production, including the modified Hodge test (MHT) and ethylenediaminetetraacetic acid (EDTA) test. The detection of carbapenemase genes was performed by multiplex and simple polymerase chain reaction (PCR).

Results: A total of 140 strains or 100% of isolates contained OXA-51 and ISbA1 sequences, 89% contained OXA-23 and OXA-58 sequences, and 1% contained OXA-24 sequence. The MBL genes were predominated by Verona integron-encoded metallo-β-lactamase (VIM) (56%), followed by Seoul imipenemase (SIM) (39%), German imipenemase (GIM) (37%), São Paulo metallo-β-lactamase (SPM) (13%), imipenemase (IMP) (11%), and New Delhi metallo-β-lactamase (NDM) (4%). Guyana extended-spectrum β-lactamase (GES) was not found in any isolation.

Conclusion: Our study shows a high frequency of carbapenem resistance in *Acinetobacter baumannii*, as it reports a high molecular diversity of carbapenemase-encoding genes, mainly dominated by the carbapenemase ISaba1/OXA-23, which represents an emerging threat in our hospital.

## Introduction

*Acinetobacter baumannii* (*A. baumannii*) is a gram-negative coccobacillus, opportunistic pathogen that has recently spread worldwide causing epidemics of nosocomial infections, mainly due to its remarkable resistance to antibiotics. Long-term survival in the environment associated with the emergence of drug resistance increases the likelihood of nosocomial transmission. Immunocompromised patients are often the target of this pathogen [1].

The overuse of antibiotics, especially carbapenems, leads to the emergence of multidrug resistance to antibiotics that further increase the intrinsic resistance of *A. baumannii*, especially in intensive care units, and limits treatment options for infections caused by these bacteria [2].

*Acinetobacter baumannii* is considered one of the most problematic species because it is resistant to diverse antibiotics of different classes, including carbapenems [3]. Carbapenem resistance is mediated by several mechanisms, including an expulsion pump, decreased membrane permeability by loss of outer membrane porins (OMPs) due to the downregulation of their synthesis, and carbapenemase production [4]. The most described carbapenem resistance mechanism in *A. baumannii* is enzymatic degradation by carbapenemase enzymes, in particular oxacillinases (OXAs) and metallo-β-lactamases (MBLs). These enzymes are encoded by mostly plasmid genes [5].

The most described oxacillinases (OXAs) in *A. baumannii* include many subgroups: intrinsic OXA-51, also considered as a marker gene for *A. baumannii*, and other acquired enzymes of OXA-23 types, OXA-58 and OXA-24 [1].

MBLs are enzymes with carbapenemase activity that threaten clinicians, on the one hand, because of their ability to efficiently hydrolyze carbapenems and, on the other hand, because of the high degree of resistance of the bacteria that produce them. Various types of MBLs have been reported worldwide in strains of *A. baumannii*, including imipenemase (IMP), Verona integron-encoded metallo-β-lactamase (VIM), New Delhi metallo-β-lactamase (NDM), São Paulo metallo-β-lactamase (SPM), Seoul imipenemase (SIM), and German imipenemase (GIM) [6].

The detection of different carbapenemases is important to determine the severity of the problem and to guide the application of antibiotic stewardship guidelines to limit the development of other carbapenem-resistant variants in the *A. baumannii* community.

Numerous studies on carbapenem resistance in *A. baumannii* have been reported worldwide, mainly concerning carbapenemase production. In North Africa, more specifically in Morocco, the presence of the carbapenemase gene in *A. baumannii* has been detected. Except for sporadic studies [7,8], we are not aware of any other studies on the characterization of carbapenemase genes in *A. baumannii* in Morocco.

In this regard, the aim of this study is to characterize the molecular profile of carbapenem-resistant *A. baumannii* among clinical isolates in Morocco at Centre Hospitalier Universitaire (CHU) Hassan II in Fez.

## Materials and methods

This cross-sectional study was conducted at the bacteriology laboratory of Centre Hospitalier Universitaire (CHU) Hassan II in Fez, Morocco, between November 2018 and July 2019. Ethical approval has been granted by the ethical committee of CHU Hassan II University Hospital, Fez. Inpatient specimens collected included blood, catheters, urine, endotracheal secretions from sputum, wound swabs, and cerebrospinal fluid (CSF).

Culture and identification

Samples were inoculated on blood agar, chocolate agar, and MacConkey agar and incubated for 24 hours at 37°C. The identification was based on phenotypic methods, Api 20 gallery (bioMérieux, Marcy-l'Etoile, France), and Phoenix 100 gallery (Becton Dickinson Co., Sparks, MD, USA). Polymerase chain reaction (PCR) confirmed the identification of the OXA-51 gene specific to *A. baumannii*.

Antimicrobial susceptibility

Antibiotic susceptibility, including carbapenems, of clinical isolates was tested using a standard Müller-Hinton agar disc diffusion technique using oxoid disc of piperacillin (PEP), ticarcillin (TIC), piperacillin/tazobactam (PTZ), ceftazidime (CAZ), imipenem (IMP), gentamicin (GM), amikacin (AK), tobramycin (TOB), and ciprofloxacin (CIP), according to the 2018 Clinical and Laboratory Standards Institute (CLSI) guidelines.

Phenotypic detection of carbapenems

Modified Hodge Test (MHT)

The modified Hodge test (MHT) was carried out according to the method described by Lee et al. [9]. One-tenth of the 0.5 McFarland suspension of carbapenem-susceptible *Escherichia coli* (*E. coli*) was aseptically buffered on sterile Müller-Hinton agar. An imipenem disc (10 µg) was placed in the center of the plate. A straight line was drawn from the inside to the outside of the plate; each stump has been streaked. A *Klebsiella pneumoniae* strain was used as a positive control. The dishes were then incubated for 18-24 hours at 37°C and then inspected to detect a space similar to a clover leaf in the inhibition zone of the carbapenem layer at the crossing point of the tested strain and *E. coli*.

Double-Disc Synergy Test (DDST)

DDST was carried out according to the method described by Lee et al. [9] to detect MBL enzymes. A young culture was diluted to 10^5^ CFU/mL and seeded on Müller-Hinton agar with a cotton swab. Two IMP (oxoid) discs were placed on the surface of the agar at 4-5 cm from each other (center to center). Then, 10 mL of 0.5 M ethylenediaminetetraacetic acid (EDTA) was added to one of the two discs. The dish was incubated for 18-24 hours. The presence of a synergistic inhibitory zone was considered positive for MBL.

E-Test MBL

E-test MBL strips (bioMérieux, Solna, Sweden) containing one side of imipenem (4-256 µg/mL) and imipenem (1-64 µg/mL) combined with a fixed concentration of EDTA were used to detect MBL. It is rated according to the manufacturer’s instructions. Minimum inhibitory concentration (MIC) levels ≥ 8 for both imipenem and imipenem responses to EDTA are indicative of MBL production.

Molecular detection of carbapenemase genes

Total DNA Extraction

Total DNA extraction was carried out using the thermal shock method. A suspension of the strains is studied in 500 µL of distilled water. After boiling the suspension for 10 minutes, it is immediately placed on the ice for two minutes and then centrifuged for 10 minutes at 14,000 rpm. We recovered 300 µL of supernatant in a new 1.5 mL Eppendorf tube and stored it at -20°C.

Gene Amplification by PCR

PCR amplification was carried out using specific primers (Table 1). The course of the amplification was started by preparing a PCR mix in a final volume of 50 µL. The PCR mix contains 2 µL of DNA, 5 µL of 10× PCR buffer, 2.5 mmol/µL of MgCl2, 100 µm of deoxynucleotide triphosphates (DNTP), 0.4 µL of each primer, and DreamTaq DNA polymerase. The gene amplification program of the oxacillinases OXA-23, OXA-24/40, OXA-51, and OXA-58 and the ISbA1 insertion sequence starts at 94°C for five minutes, followed by 30 cycles. Each cycle consisted of 94°C for 25 seconds, 52°C for 40 seconds, and 72°C for 50 seconds, with a final extension step (72°C for six minutes) to achieve the amplification.

**Table 1:** List of primers used in this work VIM: Verona integron-encoded metallo-β-lactamase, NDM: New Delhi metallo-β-lactamase, IMP: imipenemase, GIM: German imipenemase, GES: Guyana extended-spectrum β-lactamase, SIM: Seoul imipenemase, SPM: São Paulo metallo-β-lactamase

Gene	Primer	Sequence 5’3’	Size (bp)	Reference	
					OXA-23	Oxa-F	5’- GATCGGATTGGAGAACCAGA-3’	501	[10]	
Oxa-R	5‘-ATTCTTGACCGCATTTCCAT-3’								
OXA-51	Oxa -F	5’-TAATGCTTTGATCGGCCTTG-3’	353						
	Oxa-R	5’-TGGATTGCACTTCATCTTGG -3’							
OXA-24	Oxa-F	5’-GGTTGTTGGCCCCCTTAAA-3’	246						
	Oxa-R	5-AGTTGAGCGAAAAGGGGATT-3’							
OXA-58	Oxa-F	5’-AAGTATTGGGGCTTGTGCTG-3’	599						
	Oxa-R	5’-CCCCTCTGCGCTCTACATAC-3’							
VIM	VIM-F	5’-GATGGTGTTTGGTCGCATA-3’	390						
	VIM-R	5’-CGAATGCGCAGCACCAG-3’							
NDM	NDM-F	5’-GGTTTGGCGATCTGGTTTTC-3’	621						
	NDM-R	5’-CGGAATGGCTCATCACGATC-3’							
IMP	IMP-F	5’-GGAATAGAGTGGCTTAAYTCTC-3’	188						
	IMP-R	5’-CCAAACYACTASGTTATCT-3’							
GIM	GIM-F	5′-TCGACACACCTTGGTCTGAA-3′	477						
	GIM-R	5′-AACTTCCAACTTTGCCATGC-3′							
GES	GES-F	5’- AGTCGGCTAGACCGGAAAG-3’	399						
	GES-R	5’- TTTGTCCGTGCTCAGGAT-3’							
SIM	SIM-F	5′-TACAAGGGATTCGGCATCG-3′	570						
	SIM-R	5′-TAATGGCCTGTTCCCATGTG-3′							
SPM	SPM-F	5'-AAAATCTGGGTACGCAAACG-3’	271						
	SPM-R	5'-ACATTATCCGCTGGAACAGG-3’							
ISbA1	ISbA1-F	5’- CACGAATGCAGAAGTTG-3’	520						
	ISbA1-R	5’- CGACGAATACTATGACAC-3’							

The SIM, IMP, SIM, GIM, and SPM gene amplification program starts with a temperature of 94°C for five minutes, followed by 30 cycles. Each cycle consisted of 94°C for 25 seconds, 56°C (58°C for the NDM gene) for 40 seconds, and 72°C for 50 seconds, with a last extension step (72°C for six minutes) to achieve the amplification [10]. The PCR products were separated on agarose gel (1.5%) for one hour.

Sequence Analysis

The PCR products positive for the MBL genes, in particular VIM, NDM, and SPM, were purified with the BigDye kit and sequenced. The analysis of the nucleotide sequence was carried out by Basic Local Alignment Search Tool (BLAST) at National Center for Biotechnology Information (NCBI) (www.ncbi.nlm.nih.gov/BLAST).

## Results

Of 150 *Acinetobacter baumannii*-positive samples, 140 strains showed carbapenem resistance, with a frequency of 93%. The distribution of *A. baumannii* isolates by sampling site showed a predominance of protected distal specimens (PDS) at 46%, followed by blood cultures and catheters in equal proportions at 19%, wound swabs at 10%, urine at 4%, and CSF at 3% (Table 2).

**Table 2:** Distribution of isolates of A. baumannii by sampling site and department CFS: cerebrospinal fluid

Specimen	Total (number (%))	Origin	
		Intensive care unit (number (%))	Other department (number (%))
Protected distal specimens	64 (46%)	64 (46%)	0 (0%)
Blood	26 (19%)	24 (17%)	2 (11%)
Catheters	26 (19%)	24 (17%)	2 (11%)
Wound swabs	14 (10%)	2 (1%)	12 (67%)
Urine	6 (4%)	4 (3%)	2 (11%)
CSF	4 (3%)	4 (3%)	0 (0%)

The modified Hodge test (MHT) was positive for 71% of the strains tested, while the double-disc synergy test (DDST) to detect MBL production was positive for 76% of strains tested. In addition, E-test MBL for the detection of MBL production was positive for 83% of bacterial isolates (Table 3).

**Table 3:** Positivity rate of phenotypic tests for the detection of carbapenems MHT: modified Hodge test, DDST: double-disc synergy test, MBL: metallo-β-lactamase

Phenotypic test	Positive (number)	Negative (number)	Rate of positivity (%)
MHT	100	40	71%
DDST	106	34	76%
E-test MBL	116	24	83%

For the molecular detection of genes encoding different carbapenemases and insertion sequences ISbA1, the OXA-51 gene and ISbA1 sequence were detected in all isolates (100%), and OXA-23, OXA-58, and OXA-24 were detected in 89%, 7%, and 1% of the isolates, respectively. MBL genes were predominated by VIM (56%), followed by SIM (39%), GIM (37%), SPM (13%), IMP (11%), and NDM (4%). GES was not found in any isolation (Table 4).

**Table 4:** Distribution of oxacillinase and MBL genes and the ISbA1 sequence in isolates of A. baumannii MBL: metallo-β-lactamase, IMP: imipenemase, VIM: Verona integron-encoded metallo-β-lactamase, SPM: São Paulo metallo-β-lactamase, NDM: New Delhi metallo-β-lactamase, SIM: Seoul imipenemase, GIM: German imipenemase, GES: Guyana extended-spectrum β-lactamase

Gene	Positive (number)	Negative (number)	Positivity rate (%)
OXA-51	140	0	100%
OXA-23	124	16	89%
OXA-24	2	138	1%
OXA-58	10	130	7%
ISbA1	140	0	100%
IMP	16	124	11%
VIM	78	62	56%
SPM	18	122	13%
NDM	6	134	4%
SIM	54	86	39%
GIM	52	88	37%
GES	0	140	0%

## Discussion

Our study showed a predominance of protected distal specimens (PDS) at 46%, followed by blood cultures and catheters at 18.7%. There is a high prevalence of *A. baumannii* strains in bronchopulmonary samples, according to several studies [11]. According to many studies, mechanical ventilation is associated with a high incidence of *A. baumannii* pneumonia, which results in prolonged stays in intensive care units and rapid antibiotic resistance, with a high mortality rate of 45.6%-84.3% [11,12]. The predominance of these infections has been confirmed in many studies [13,14]. Various risk factors associated with *Acinetobacter* infection in these services may explain this, including immunocompromised patients, longer stays, invasive devices, and the use of broad-spectrum antibiotics. This bacterium can be contaminated frequently and transmitted from healthcare workers to the environment through reservoirs and hands [12].

In our study, the frequency of infection with carbapenem-resistant *A. baumannii* was higher (93%) in our hospital. This rate in the intensive care unit is high compared to other units. This confirms that *A. baumannii* is an emerging germ in intensive care [7].

A similar high rate of carbapenem resistance was observed in Morocco (100%) recently by Uwingabiye et al. [15]. It is possible that this high rate is linked to the overuse of carbapenems and third-generation cephalosporins. The selection of strains of *A. baumannii* resistant to carbapenems has been linked to the pressure exerted by third-generation cephalosporins [16].

Carbapenem resistance can be traced back to several mechanisms. However, the production of MBL and oxacillinase remains the most common mechanism in *A. baumannii* [1].

Among *A. baumannii* carbapenemases, oxacillinases are by far the most common [17]. Furthermore, in our isolates, OXA-51, OXA-23, and ISbA1 coexist, explaining their resistance to all antibiotics, including carbapenems. As a matter of fact, the molecular characterization of isolates resistant to carbapenems reveals that in all isolates, in addition to the intrinsic gene OXA-51, the ISbA1 gene is required to overexpress carbapenemase production [18].

We found the coexistence of OXA-51 and OXA-23 genes in 83% of clinical isolates, which is consistent with other studies [19]. As the OXA-23 gene is plasmid-encoded, it may be due to the horizontal spread of plasmid-bearing plasmids that OXA-23 is commonly found in our hospital and in other Moroccan regions [7].

MBLs can hydrolyze all β-lactams except monobactam (e.g., aztreonam). EDTA inhibits their activity, but clavulanic acid does not [20]. We found a diversity of genes responsible for encoding MBLs for these enzymes, thus explaining the high resistance rates of our isolates due to MBL enzymes. Indeed, we detected the gene coding for the VIM in 56% of strains, a higher rate than in other studies at 5.7% [20] and 7% [21]. However, this is slightly consistent with that reported by PeshattiWar et al., the rate being noted at 62.5% [22]. Moreover, we detected the gene coding for SIM in 39% and the gene encoding for GIM in 37%. These three enzymes predominate MBLs in this study.

The SPM gene was detected in 13% of the isolates. Knowing that, the primary report of metallo-β-lactamase from São Paulo was reported in Brazil in an isolate of *Pseudomonas aeruginosa *(*P. aeruginosa*), then this enzyme spread throughout Brazil [23], and its first report in Morocco was made by a study already done by our team and published [24].

The IMP gene was found in 11% of the strains. This is a low rate compared to other studies that reported a rate of 95% [25].

The IMP and VIM genes are found along with other resistance genes on integrons associated with transposons. This facilitates translocation and rapid horizontal spread between chromosomes and plasmids [25].

With regard to the NDM-type enzyme, strains of *A. baumannii* hosting these enzymes are highly observed all over the world, particularly in eastern countries [26]. They have been detected in North Africa, in Algeria [27] and Morocco [15]. The NDM gene was found on several plasmids [28]. The location of NDM on the plasmid facilitates rapid horizontal spread of gram-negative bacteria by conjugation [28]. NDM-1 rates in our study are similar to those in Egypt and Ghana at 8% and 19%, respectively [29].

Due to a lack of resources, this study is not able to sequence many strains nor do a clonality study using pulsed-field gel electrophoresis (PFGE) to determine if our extremely resistant strains are kin.

## Conclusions

Our hospital is experiencing an alarming amount of carbapenem resistance in strains of *A. baumannii*. The high genomic plasticity of this bacterium and its horizontal and clonal design can be explained by the genetic diversity observed in our strains. The control of nosocomial carbapenem-resistant *A. baumannii* infection and the prevention of dissemination of resistance genes between endemic nosocomial pathogens require local molecular surveillance.
